# Does Age Influence the Outcome of Lower Limb Non-Union Treatment? A Matched Pair Analysis

**DOI:** 10.3390/jcm8091276

**Published:** 2019-08-22

**Authors:** Michael Tanner, Wasilios Vlachopoulos, Sebastian Findeisen, Matthias Miska, Julian Ober, Saskia Hagelskamp, Gerhard Schmidmaier, Patrick Haubruck

**Affiliations:** HTRG—Heidelberg Trauma Research Group, Center for Orthopedics, Trauma Surgery and Spinal Cord Injury, Trauma and Reconstructive Surgery, Heidelberg University Hospital, D-69118 Heidelberg, Germany

**Keywords:** non-union, bone healing, older adults, masquelet-therapy, non-union therapy, diamond concept

## Abstract

Background: Fractures in elderly patients are common and have severe implications on a socioeconomic level, as musculoskeletal integrity and competence is crucial for independence. Changes in both composition and biology of bones during aging potentially affect fracture healing adversely. The current study sought to determine the influence of age on the outcome of non-union therapy of atrophic and hypertrophic non-unions based on the “diamond concept”, as well as to evaluate the well-known risk factors impairing bone healing. Patients and Methods: All medical records, operative notes, lab data, and radiological imaging of patients that received surgical treatment of both atrophic and hypertrophic non-unions of the femur or tibia between 1 January 2010 and 31 December 2016 were thoroughly reviewed and analyzed. Patients who participated in our standardized follow-up for at least 12 months were included into a database. Patients older than 60 years were matched with patients younger than 60 based on five established criteria. The study was approved by the local ethics committee (S-262/2017). According to our inclusion criteria, a total of 76 patients older than 60 years were eligible for analysis. Via matching, two groups were formed: study group (SG; >60 years; *n* = 45) and control group (CG; <60 years; *n* = 45). Results: Twelve months subsequent to treatment, the consolidation rate was equivalent in both groups (SG: 71% vs. CG: 67%). The consolidation for all patients before matching was 73%. The clinical results for the complete collective were no pain or pain with high or medium strain for 62.5%, whereas 29.6% had pain with low strain or constant pain. 7.87% had no pain levels given. Logistic regression modeling showed no influence of age >60 years on radiological or clinical outcome, whereas a significant negative correlation was revealed between patients aged 40–49 years and radiological non-union consolidation (b = −1.145 and *p* = 0.048). In addition, diabetes had a negative influence on non-union therapy (b = −1.145 and *p* = 0.048). As expected, the clinical outcome correlated significantly with the radiological outcome (*p* < 0.001). Conclusion: Surgeons should optimize both modifiable risk factors such as diabetes mellitus, as well as surgical treatment in order to achieve the best possible outcome in elderly patients. Elderly patients benefit from osseous consolidation by enabling and maintaining musculoskeletal competence due to the close correlation between clinical and radiological outcome. Advanced age alone does not negatively influence the outcome of non-union therapy and should, therefore, not be considered a risk factor. In contrast, patients in their fifth decade suffering from lower limb non-unions should be considered as high-risk patients and treatment should be modified accordingly.

## 1. Introduction

Fractures in elderly patients are common, and it is estimated that the lifetime risk of any fracture at the age of 60 is 47% for women and 22% for men [1]. Osteoporosis is characterized by the loss of bone mass and bone strength, predisposing the affected patients to low-energy or fragility fractures [2]. About 90% of fragility fractures occur in patients 60 years or older [2], and as the number of elderly is rising, fracture rates are expected to increase accordingly worldwide [2]. Fractures in elderly patients have severe implications not only clinically, but also on a socioeconomic level, as musculoskeletal competence and integrity is crucial for independence, continued mobility and function, maintained quality of life, and social participation [3].

Fracture healing is a complex process, and multiple cellular and molecular factors need to act concertedly in order to achieve proper osseous consolidation [4]. Additionally, risk factors for non-union are multifaceted, including intrinsic (host factors and biological bone factors) and extrinsic (mechanical factors, infection and utilized treatment) aspects [5]. Elderly patients exhibit changes in bone composition, cell differentiation potential, decreased osteoblastic response to mechanical stimuli, and insufficient vascularization, all potentially adversely affecting fracture healing [4,6]. Non-union of a fracture prolongs the necessary treatment and is associated with severe limitations in the patients’ quality of life [7]. Evidence exists that these complications are more common in the elderly [8,9]. Next to osseous consolidation, successful non-union treatment in elderly patients should focus on decreasing the associated comorbidities and enabling patient independence with continued mobility and function [10,11]. The “diamond concept” is widely accepted for its principles in non-union therapy [12] and treatment based thereupon has shown good clinical results in various long-bone non-unions [13,14,15]. In particular, this concept has been established as a conceptual framework for successful bone repair response [16] and gives the mechanical stability and biological environment equal importance. According to this, mandatory factors that have to be provided through non-union therapy for successful bone repair are vascularity, osteoinductive factors, osteogenic cells, osteoconductive matrix, and mechanical stability [16]. The influence of age on the outcome of non-union therapy; however, remains unclear. In this study we, therefore, sought to determine the effect of age on the outcomes of one- or two-step non-union therapies of atrophic and hypertrophic non-unions based on the “diamond concept” utilizing a matched-pair analysis.

## 2. Patients and Methods

### 2.1. Study Design

The current study was designed as a retrospective matched-pair analysis based on a clinical database and was performed in concordance with the Declaration of Helsinki. Before the commencement of the study, approval from the ethics committee of the University of Heidelberg (S-262/2017) was obtained. Hereafter all medical records, operative notes, lab data and radiological imaging of patients that received surgical treatment of lower limb non-unions (i.e., of the femur or the tibia, both atrophic and hypertrophic) between 1 January 2010 and 31 December 2016 were analyzed. In particular, diaphyseal non-unions in the femur and the tibia in addition to patients who were surgically treated based on the “diamond concept” in our institution and participated in our standardized follow-up for at least 12 months were included in the current study. Operative treatment for non-union therapy strictly followed recommendations of the “diamond concept”. Depending on the type of non-union, a one-step (34 patients) or two-step procedure (56 patients) was applied. These were evenly distributed among the 90 patients of the control and age groups of 45 patients each. Patients treated with a two-step procedure received Masquelet-therapy [17]. Consequently, both atrophic and hypertrophic non-unions were included. Exclusion criteria were follow-up shorter than 12 months, joint-replacement therapy, treated non-unions in other anatomical locations than the lower limb, the existence of systemic inflammatory diseases, malignancy, as well as long-term use of immunosuppressive drugs. Patient data were thoroughly reviewed regarding patient characteristics, type of surgical procedure, post-operative pain, post-operative weight-bearing, mechanical stability, history of infection and radiological signs of consolidation. Patients older than 60 years were matched with patients younger than 60.

**Figure 1:**
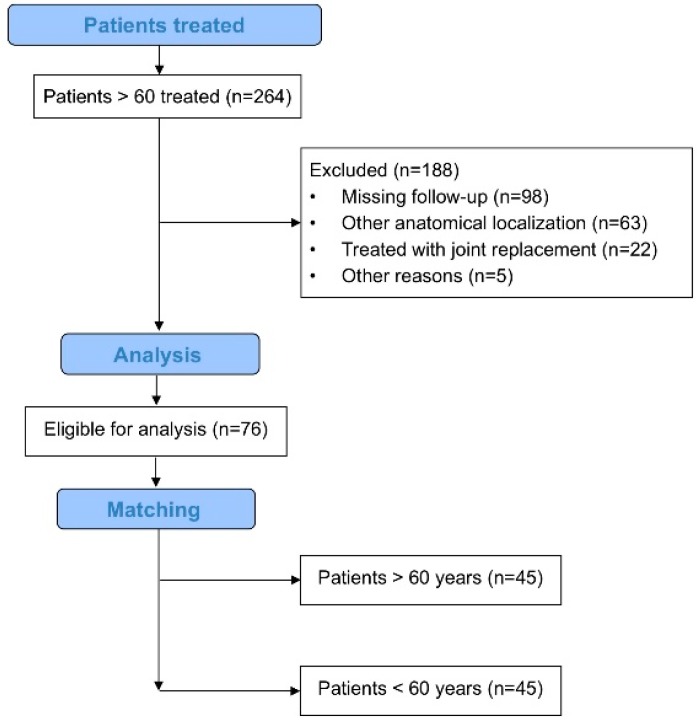
Flow chart visualizing the patient selection process.

Two-hundred and sixty-four patients older than 60 years were treated in our institution between 1 January 2010 and 31 December 2016 based on the “diamond concept”. According to the exclusion criteria, 188 patients had to be excluded from the current study (161 due to an incomplete follow-up, 22 patients were treated with a joint replacement, five patients were treated with cortisone) resulting in 76 suitable patients (Figure 1). Hereafter, eligible patients were then matched with patients younger than 60 years based on the matching criteria. We were able to assign a total of 45 matching patients to each group. Patients in the study group were an average of 67 years old, whereas patients in the control group were an average of 43 years old. The tibia was affected in 51% in both groups (femurs 49%) and 17 patients (38%) were treated with one-step surgery, whereas 28 patients (62%) were treated with Masquelet-therapy. In each group, 16 patients suffered from an infected non-union and BMP-7 (Bone Morphogenetic Protein 7) was used as an adjunct treatment in 31 cases in both groups. Additional data concerning patient characteristics is found in Table 1.

### 2.2. Matching of Patients

A total of two groups were compared in the current study:

1. Study group: patients receiving surgical treatment based on the “diamond concept” older than 60 years
2. Control group: patients receiving surgical treatment based on the “diamond concept” younger than 60 years

Matching was conducted by a clinician blinded towards clinical and radiological results; furthermore, the matcher had no further participation in the study. The matching criteria were based on previously published effective criteria [18], and a total of five criteria were used for matching of patients: sex, affected long bone, performed surgical treatment (one-step vs. two-step), smoking status and history of infection. If more than one match was found, the patient with the most similar type of non-union was chosen. According to the matching criteria, two groups (*n* = 45) could be formed of the total study patients (Table 1).

### 2.3. Surgical Technique

Depending on the type of non-union, defect size, and previous patient history (paying special attention to any history of infection), a one or two-step procedure was performed. Ultimately the type of surgery was at the discretion of the responsible surgeon. Regardless, the type of surgery, multiple tissue samples and swabs were harvested and collected during each surgery for microbial testing. In particular, for the two-step procedure, the induced membrane or Masquelet-therapy [19] was utilized. One-step treatment was used in smaller aseptic atrophic non-unions (defect size shorter than 2 cm in length) and hypertrophic non-unions. During one-step treatment, the non-union and surrounding avital soft tissue was debrided, and the resulting osseous defect filled with autologous bone graft and occasionally additional growth factors were applied (e.g., bone morphogenic protein 2 or 7; 3.3 mg BMP-7, or 4 mg BMP-2) [10,19,20,21]. Biomechanical stability was achieved by de-novo osteosynthesis using intramedullary nails or plates. The Masquelet-therapy was utilized in atrophic non-unions if any infection was suspected or if a larger defect size (>2 cm in length) was apparent. The first step of the Masquelet-therapy consisted of radical debridement of the non-union and surrounding avital soft tissue, and the resulting bone defect was filled with a polymethyl methacrylate (PMMA) spacer impregnated with gentamycin and vancomycin [13]. If an infection was detected, the first step was repeated until asepsis was achieved; hereafter, the spacer was left in-situ for a total of six weeks to ensure a fully grown Masquelet-membrane [19]. During the second surgery, the spacer was removed and the resulting bone defect was grafted with autologous spongiosa while leaving the membrane unimpaired; use of growth factors and realization of biomechanical stability was performed analogue to the one-step surgery.

Tissue samples were immediately processed after harvesting according to the standard of care of our microbiological department. Evidence of bacteria was determined as infection if obligate pathogen bacteria were detected or if more than two independent samples were positive for the same facultative pathogen bacteria [22]. Tissue processing in our microbiological department was performed as previously described [22,23].

Perioperatively 1.5 g of Cefuroxim (if no contraindication was present) was administered and continued three times a day until the results from the obtained tissue samples were received. If an infection was present, the antibiotics were modified with the help of clinical pharmacists based on susceptibility of each detected bacteria. Antibiotics were administered for a minimum of four weeks or until both the serum values of CRP (C-reactive protein) went back to normal, and the wound was fully healed. In patients receiving the two-stage therapy, the first step was repeated until asepsis was achieved. Further information regarding antibiotic treatment have been published elsewhere [22].

### 2.4. Follow-Up

All patients receiving surgical treatment of non-unions in our institution were invited to attend our specialized follow-up program designed to guide the patient through the initial and crucial phase of bone regeneration and allow early assessment of both possible complications and clinical and radiological outcome. Therefore, postoperative radiological and clinical follow-up consultations were scheduled at two days, one and six weeks, as well as 3, 6 and 12 months, and then annually. Only patients that attended every follow-up consultation in the first year were included in the current study to ensure completeness of the data.

### 2.5. Analysis of Radiological and Clinical Outcome

The clinical and radiological outcome was determined 12 months subsequent to the final surgical non-union treatment. During each follow-up consultation, patients were independently examined by two experienced and blinded trauma surgeons. The clinical outcome was evaluated based on pain associated with weight-bearing, clinical signs of mechanical stability, as well as subjective patient history. Pain associated with weight-bearing was further stratified into five categories (no pain associated with weight-bearing, pain associated with high physical strain, pain associated with medium physical strain, pain associated with low physical strain, constant pain without physical strain). Available X-rays and CT-scans (only if medically indicated) were evaluated independently by two different experienced and blinded trauma surgeons, and non-unions were evaluated as consolidated based on the bridging of three out of four cortices. The generated data was imported into a database for further statistical analysis.

### 2.6. Statistics

Statistical analysis was performed utilizing SPSS for Windows 10 (SPSS Inc., Chicago, IL, USA). The Chi-square test was used to evaluate categorical and statistically significant differences between groups, location shifts between groups via non-parametric test methods (Mann–Whitney U-Test). Significant changes within both groups were analyzed via Wilcoxon signed-rank test for paired samples. Correlation analyses were conducted between all variables and the predictive performance of any logistic regression model was evaluated based on the area under the curve (AUC) of the respective ROC (receiver operating characteristic) curve. In particular, factors that were included into the regression analysis were the type of non-union, age, utilized type of BMP, method of osteosynthesis and diabetes. Continuous variables are expressed as absolute mean concentrations ± SD (standard deviation) and the level of significance (α) was set at 5%.

## 3. Results

### 3.1. Analysis of Non-Union Therapy Outcome

**Figure 2:**
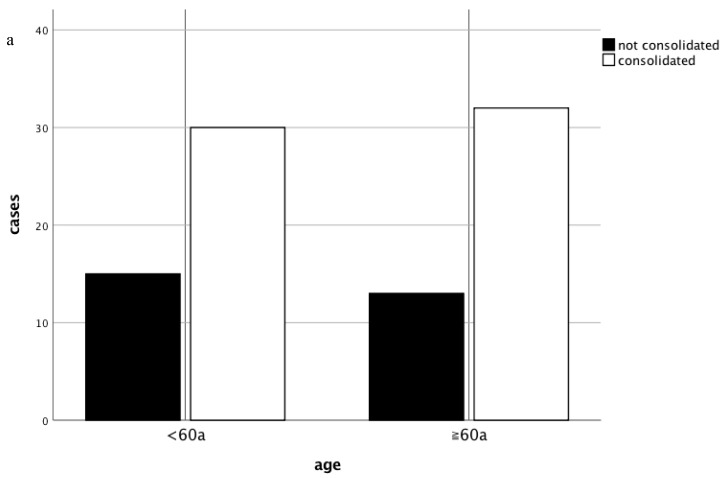

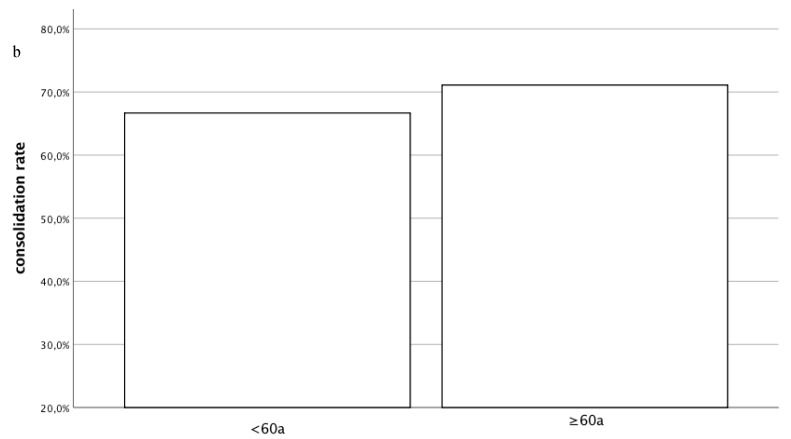
Radiological outcome of non-union treatment based on the “diamond concept”. The consolidation subsequent to the non-union therapy in respect to patients’ age is shown in absolute numbers (**a**) and percentage (**b**).

**Figure 3:**
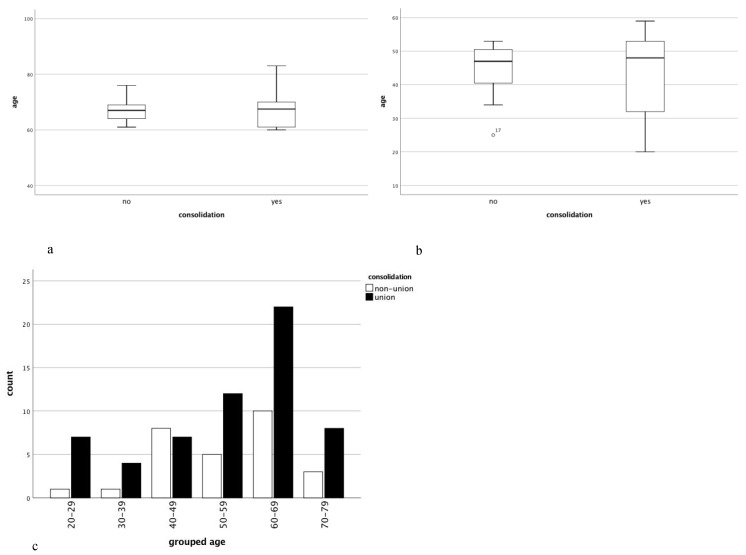
Radiological outcome in context with the age of patients. Visualization of the age of patients stratified based on consolidation after treatment is shown using box plots for the study group (**a**) and control group (**b**). Whiskers indicate minimum and maximum, the band inside the box indicates median. Furthermore, consolidation in respect to patients’ age was subdivided into decades is shown (**c**).

Twelve months subsequent to the non-union treatment, 32 patients older than 60 years (71%) showed proper osseous consolidation compared to 30 patients in the control group (67%) (*p* > 0.05) (Figure 2a,b). In 13 patients (29%) from the study group non-union treatment based on the “diamond concept” failed. These patients were an average of 71 ± 7 years old and 46% were female (Figure 3a). In 7 cases the femur was affected (54%) and 8 patients received Masquelet-therapy (62%). In comparison 15 patients (33%) from the control group failed to respond to the non-union treatment averaging 45 ± 8 years (Figure 3b). 7 patients (47%) were male and in 4 cases the tibia (27%) was affected. It is noteworthy that 80% of patients from the control group that failed to show osseous consolidation had received Masquelet-therapy. Furthermore, 87% of patients from the control group and 92% of patients from the study group in which the treatment failed had received adjunct application of rhBMP-7. Only 1 patient treated with BMP-2 (study group) failed to consolidate. Additional information regarding the characteristics of non-responders can be found in Table 2.

### 3.2. Analysis of Risk Factors’ Influence on Outcome

Logistic regression modeling regarding the influence of common risk factors on radiological and clinical outcome was performed.

#### 3.2.1. Radiological Outcome

Our study showed no influence of age >60 years on radiological outcome, regardless of the applied method of non-union treatment (see Figure 2). The overall consolidation rate before matching or subdivision into groups was equivalent to that of our prior findings at 73%. To further investigate the influence of age on the outcome of therapy, patients were stratified according to their age into 10-year periods. Interestingly, regression modeling revealed only a significant negative correlation between patients aged 40–49 years and non-union consolidation (b = −1.145 and *p* = 0.048) (Figure 3c). Adjunct therapy with BMP-7 correlated negatively with radiological consolidation (b = −2.214) in all patients with a borderline significance (*p* = 0.052). Despite the small number of patients suffering from diabetes, the analysis revealed a negative correlation with consolidation in the total collective (b = −1.145 and *p* = 0.048). In patients older than 60 years, the anatomical localization did not influence their outcome (b = −0.279 and *p* = 0.672); however, in patients younger than 60 years, the outcome of the treatment had a strong negative correlation with non-unions of the femur (b = −1.558 and *p* = 0.025).

Active smoking correlated negatively with radiological outcome in both groups to a non-significant extent. Regarding other established risk factors, neither patient gender, biological status of the non-union, nor the method of osteosynthesis had an influence on the radiological outcome.

#### 3.2.2. Clinical Outcome

**Figure 4:**
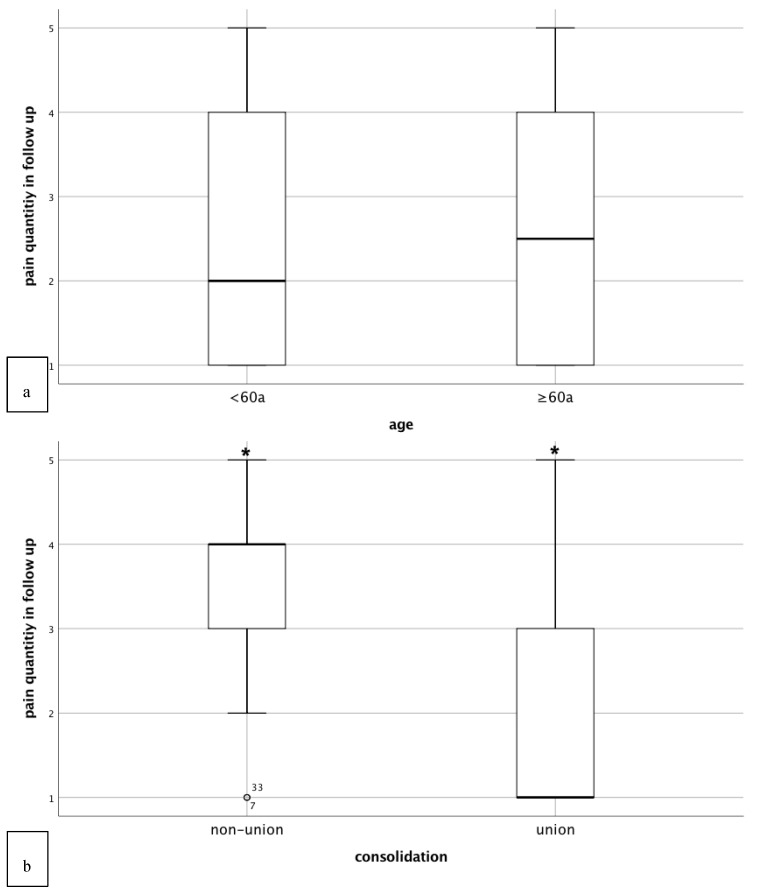
Clinical outcome of non-union treatment based on the “diamond concept”. Here pain associated with weight-bearing in respect to patients’ age (**a**) and consolidation status (**b**) is shown using box plots. Whiskers indicated minimum and maximum, band inside box indicates median.

In our complete collective prior to matching, the clinical results for the complete collective were no pain or pain with high or medium strain for 62.5% of patients. 29.6% had constant pain or pain with low strain. 7.87% had no pain levels given. Age had no influence on the clinical outcome of non-union therapy (Figure 4a). In particular, as expected, pain associated with weight-bearing significantly correlated with the radiological outcome regardless of the age of patients (*p* < 0.001) (Figure 4b). Similar to the radiological outcome, diabetes mellitus correlated significantly with an impaired clinical outcome in all patients (b = −2.107 and *p* = 0.035). Besides these, no other established risk factor (smoking, gender, type of osteosynthesis, biological status of non-union) correlated with the clinical outcome of non-union therapy. Distribution of pain associated with weight-bearing was similar in patients, both older and younger than 60 years (Table 3).

## 4. Discussion

In the current study, we sought to determine the influence of age onto the radiological and clinical outcomes subsequent to non-union therapy based on the “diamond concept”. In addition, we strove to analyze established risk factors known to impair bone healing regarding their impact onto the consolidation of non-unions and compared the results between patients older and younger than 60 years.

### 4.1. Influence of Age on Radiological Outcome of Non-Union Therapy

Review of current literature revealed some evidence that “elderly” patients are at risk for delayed fracture healing or non-union of a fracture; whereby the influence of age on the development of non-unions remains controversial [11]. Foulke et al. postulated that increased age leads to physiological changes, leaving “elderly” patients more susceptible to fractures and subsequent healing complications [11]. In particular, delayed fracture healing in “elderly” patients might be the result of an altered capacity for mesenchymal progenitor stem cell differentiation, impaired angiogenesis and reduced levels of growth factors [11,24]. Zura et al. reported the non-union rate after fracturing in 47,437 patients [25]. Interestingly, according to their results, the patients developing non-unions were younger than patients that healed normally and patients over 65 years had a lower risk-adjusted non-union rate [25]. Mills et al. investigated the relative risk of developing a long-bone non-union in another large adult population [5]. Due to the utilization of a national database involving 5000 non-unions, the authors were able to provide robust estimates of non-union risk. Interestingly, despite the fact that the number of fractures increased with age, the number of non-unions did not. In particular, the rate of fracture non-union was highest in patients aged 30 to 44 years and 2.5 times higher than in those aged >75 years [5]. The results from our study extend the compelling findings from Mills and Zura et al. In particular, the outcome of non-union therapy in patients older than 60 years of age was equivalent when compared to younger patients, and an impaired outcome of non-union therapy was only associated with patients between the age of 40 and 49. These results are supported by Hernandez et al. [26] showing that patients between 65 and 79 years of age were less likely to undergo healing complications than younger patients [26]. This might be due to more high-impact fractures in younger patients negatively affecting bone healing [11].

Factors hampering osseous consolidation could be subdivided into factors associated with mechanical stability and factors associated with poor fracture biology [27]. Among those risk factors, diabetes mellitus is known to increase fracture risk and cause postoperative complications, such as non-unions, infections and necessary reoperations [25,28,29]. Although the influence of diabetes mellitus with bone healing complications has been well documented, there is no robust evidence regarding its influence on the outcome of non-union therapy. As expected, the results from the current study suggest a negative influence of diabetes onto the outcome of non-union therapy regardless of age. According to Shibuya et al., hemoglobin A1c levels >7% and peripheral neuropathy were associated with impaired bone healing in diabetic patients [29]. Thus, treating surgeons should take the influence of diabetes mellitus into account and should reduce hemoglobin A1c levels <7% if possible.

In our literature review, only two other studies were identified investigating the influence of age onto the outcome of non-union therapy. Taormina et al. investigated the effect of age on clinical and functional outcome following non-union surgery in 288 patients [27]. They concluded that advanced age was generally not associated with poorer outcome [27]. Another study by Brinker et al. analyzed the functional outcome utilizing the Ilizarov method for tibial non-unions in elderly patients [30]. Despite their advanced age, all patients’ tibial non-unions healed. The current study distinguishes itself from these two other studies by utilizing a matched pair analysis and assessing further risk factors. Thereby, the risk of possible biases confounding the results of the current study is reduced, leaving age as the only relevant differing patient characteristic.

Our literature review revealed only one other study evaluating the clinical effectiveness of BMP-7 in long-bone non-union treatment of elderly adults. Murena et al. analyzed the outcome of humeral shaft non-union therapy using BMP-7 in two older adults [31]. They reported BMP-7 to be a safe and effective treatment option. In contrast, our data showed that adjunct therapy with BMP-7 was associated with failure of the treatment thereby indicating an inferior or adverse clinical impact (87% of patients from the control group and 92% of patients from the study group that did not respond to the therapy were treated with BMP-7). Known risk factors associated with compromised bone healing have been extensively studied, and the role of medical comorbidities, age, gender, and smoking have been highlighted [27,32]. Data from the current study were analyzed using multiple linear regression models, and our results indicated smoking as a risk factor regarding bone healing [32]. Smoking correlated negatively with radiological consolidation in patients, both younger and older than 60 years. Interestingly, there was no correlation between gender of patients, biological status of non-unions, or method of osteosynthesis and osseous consolidation. This might be due to the applied “diamond concept” causing an enhanced local microenvironment favorable for bone healing, and thereby, nullifying certain risk factors for regular bone healing [12,15].

### 4.2. Influence of Age on Clinical Outcome of Non-Union Therapy

Pain-free musculoskeletal competence is crucial for independence, continued mobility, and function, as well as maintaining quality of life and participation in society [3]. Therefore, clinical outcome was evaluated based on pain associated with weight-bearing. Despite the potential difficulties for elderly patients to participate in physical therapy subsequent to treatment and the possible consecutive implications on their overall mobility and clinical outcome when compared to younger patients [27], elderly patients showed a good clinical outcome. In particular, our data showed that on average, patients older than 60 years only experienced pain during high physical strain, allowing patients continued mobility and function and participation in activities of daily living. As expected, regression modeling revealed that clinical and radiological outcome correlated significantly regardless of patient age. Thereby, the results of the current study emphasize the necessity of achieving osseous consolidation in older adults to reduce pain associated with weight-bearing and increase mobility and function.

### 4.3. Limitations

There are several limitations to this study. Despite the large number of patients treated in our institution, only a small number of patients were able to be included in the current study due to the strict inclusion and exclusion criteria as well as the matched-pair design. We acknowledge that power might be lacking; nonetheless, we are unaware of any other studies presenting data pertaining to this question at all, let alone in such numbers. Nonetheless, due to the fact that other evidence regarding the treatment of non-unions in elderly patients is missing, we feel that the results of the current study add important knowledge to the literature in the field and surgeons and clinicians should be aware of the challenges and limitations for this type of treatment. Also, a large proportion of elderly patients were lost to follow-up due to the reduction in mobility, concomitant disease and difficulties in commuting to our center, as they lived far away. Efforts to contact patients with incomplete follow-up were made and partial results obtained, but only if the full follow-up was completed were they included. Also, it might be assumed that a selection bias exists through the exclusion of patients who did not complete follow-up. These patients might have different results for consolidation. Despite various efforts to reduce the possibility of a selection bias (e.g., matching by a blinded matcher) the possibility of a selection bias influencing the results of the current study remains. In addition, clinical evaluation of the outcome of therapy was based on subjective patient information leaving the risk of an over- or underreporting.

## 5. Conclusions

In conclusion, advanced age alone should not be considered as a contraindication for non-union therapy based on the “diamond concept”. Despite osteosynthesis being potentially more challenging than joint replacement, which might allow earlier mobilization under full weight-bearing, elderly patients seem to benefit from osseous consolidation by maintaining musculoskeletal competence due to the close correlation between clinical and radiological outcome. Therefore, treating surgeons should optimize both modifiable risk factors such as diabetes, as well as surgical treatment. Definite cessation of tobacco use should be achieved preoperatively by introducing patients to effective measures to ease detoxification. Surgeons should also be aware that elderly patients are disproportionately prone to dropping out of postoperative surveillance (as we noted in our large number of lost-to-follow-up), so strategies to facilitate local rehabilitation rather than the original center should be organized. Patients in their fifth decade suffering from lower limb non-unions should be considered as high-risk patients and treatment should be modified accordingly.

## Figures and Tables

**Table 1:** Patient characteristics.

Characteristic	Group	
	<60a (*n* = 45)	≥60a (*n* = 45)
Age (years)	43 ± 11	67 ± 5
Sex		
Male	28 (62%)	28 (62%)
Female	17 (38%)	17 (38%)
Affected long bone		
Tibia	22 (49%)	22 (49%)
Femur	23 (51%)	23 (51%)
Non-union treatment		
One-Step	17 (38%)	17 (38%)
Two-Step	28 (62%)	28 (62%)
Smoking status		
yes	8 (18%)	8 (18%)
no	33 (73%)	33 (73%)
former	4 (9%)	4 (9%)
Infection		
yes	16 (36%)	16 (36%)
no	29 (65%)	29 (65%)
Bone Morphogenetic Protein used		
rhBMP-2	5 (11%)	9 (20%)
rhBMP-7	31 (69%)	31 (69%)
none	9 (20%)	5 (11%)
Diabetes		
yes	3 (7%)	7 (16%)
no	42 (93%)	38 (84%)
Method of osteosynthesis		
External fixator	0 (0%)	1 (4%)
Nail	24 (53%)	19 (42%)
Plate	21 (47%)	25 (54%)
Type of non-union		
hypertrophic	10 (22%)	3 (7%)
atrophic	35 (78%)	42 (93%)
Consolidation		
yes	30 (67%)	32 (71%)
no	15 (33%)	13 (29%)

Patient characteristics of all patients included into the study. Age, sex, affected long bone, non-union treatment, smoking status and infection were used as matching criteria.

**Table 2:** Patient characteristics of non-responder to therapy.

Characteristic	Group		
	<60a (*n* = 15)	≥60a (*n* = 13)	Total (*n* = 28)
Age (years)	45 ± 8	71 ± 7	55 ± 13
Sex			
Male	7 (47%)	7 (54%)	14 (50%)
Female	8 (53%)	6 (46%)	14 (50%)
Affected long bone			
Tibia	4 (27%)	6 (46%)	10 (36%)
Femur	11 (73%)	7 (54%)	18 (64%)
Non-union treatment			
One-Step	3 (20%)	5 (38%)	8 (29%)
Two-Step	12 (80%)	8 (62%)	20 (71%)
Smoking status			
yes	3 (20%)	4 (31%)	7 (25%)
no	11 (73%)	7 (54%)	18 (64%)
former	1 (7%)	2 (15%)	3 (11%)
Infection			
yes	9 (60%)	5 (38%)	14 (50%)
no	6 (40%)	8 (62%)	14 (50%)
rhBMP			
rhBMP-2	0 (0%)	1 (8%)	1 (4%)
rhBMP-7	13 (87%)	12 (92%)	25 (89%)
none	2 (13%)	0 (0%)	2 (7%)
Diabetes			
yes	3 (20%)	3 (23%)	6 (21%)
no	12 (80%)	10 (77%)	22 (79%)
Method of osteosynthesis			
External fixator	0 (0%)	0 (0%)	0 (0%)
Nail	8 (53%)	4 (31%)	12 (43%)
Plate	7 (47%)	9 (69%)	16 (57%)
Type of non-union			
hypertrophic	3 (20%)	1 (8%)	4 (14%)
atrophic	12 (80%)	12 (92%)	24 (86%)

**Table 3:** Pain characteristics.

Group	1	2	3	4	5
<60a (*n* = 38)	21 (47%)	3 (7%)	8 (18%)	10 (22%)	2 (4%)
≥60a (*n* = 38)	17 (38%)	4 (9%)	7 (16%)	12 (27%)	2 (4%)

Pain associated with weight-bearing stratified by group of age, **1** = no pain associated with weight-bearing, **2** = pain associated with high physical strain, **3** = pain associated with medium physical strain, **4** = pain associated with low physical strain, **5** = constant pain without physical strain.

## References

[1] Nguyen N.D., Ahlborg H.G., Center J.R., Eisman J.A., Nguyen T.V. (2007). Residual lifetime risk of fractures in women and men. J. Bone Miner. Res..

[2] Lems W.F., Raterman H.G. (2017). Critical issues and current challenges in osteoporosis and fracture prevention. An overview of unmet needs. Ther. Adv. Musculoskelet. Dis..

[3] McGuigan F.E., Bartosch P., Akesson K.E. (2017). Musculoskeletal health and frailty. Best Pract. Res. Clin. Rheumatol..

[4] Tarantino U., Saturnino L., Scialdoni A., Feola M., Liuni F.M., Tempesta V., Pistillo P. (2013). Fracture healing in elderly patients: New challenges for antiosteoporotic drugs. Aging Clin. Exp. Res..

[5] Mills L.A., Aitken S.A., Simpson A. (2017). The risk of non-union per fracture: Current myths and revised figures from a population of over 4 million adults. Acta Orthop..

[6] Dalle Carbonare L., Valenti M.T., Zanatta M., Donatelli L., Lo Cascio V. (2009). Circulating mesenchymal stem cells with abnormal osteogenic differentiation in patients with osteoporosis. Arthritis Rheumatol..

[7] Moghaddam A., Zimmermann G., Hammer K., Bruckner T., Grützner P.A., von Recum J. (2011). Cigarette smoking influences the clinical and occupational outcome of patients with tibial shaft fractures. Injury.

[8] Lu C., Miclau T., Hu D., Hansen E., Tsui K., Puttlitz C., Marcucio R.S. (2005). Cellular basis for age-related changes in fracture repair. J. Orthop. Res..

[9] Egol K.A., Koval K.J., Zuckerman J.D. (1997). Functional recovery following hip fracture in the elderly. J. Orthop. Trauma.

[10] Karger C., Kishi T., Schneider L., Fitoussi F., Masquelet A.C. (2012). Treatment of posttraumatic bone defects by the induced membrane technique. Orthop. Traumatol. Surg. Res..

[11] Foulke B.A., Kendal A.R., Murray D.W., Pandit H. (2016). Fracture healing in the elderly: A review. Maturitas.

[12] Moghaddam A., Zietzschmann S., Bruckner T., Schmidmaier G. (2015). Treatment of atrophic tibia non-unions according to ‘diamond concept’: Results of one- and two-step treatment. Injury.

[13] Schmidmaier G., Moghaddam A. (2015). Long Bone Nonunion. Z Orthop. Unf..

[14] Miska M., Findeisen S., Tanner M., Biglari B., Studier-Fischer S., Grützner P.A., Schmidmaier G., Moghaddam A. (2016). Treatment of nonunions in fractures of the humeral shaft according to the Diamond Concept. Bone Jt. J..

[15] Moghaddam A., Thaler B., Bruckner T., Tanner M., Schmidmaier G. (2017). Treatment of atrophic femoral non-unions according to the diamond concept: Results of one- and two-step surgical procedure. J. Orthop..

[16] Andrzejowski P., Giannoudis P.V. (2019). The ‘diamond concept’ for long bone non-union management. J. Orthop. Traumatol..

[17] Masquelet A.C., Obert L. (2010). Induced membrane technique for bone defects in the hand and wrist. Chir. Main.

[18] Haubruck P., Kammerer A., Korff S., Apitz P., Xiao K., Büchler A., Biglari B., Zimmermann G., Daniel V., Schmidmaier G. (2016). The treatment of nonunions with application of BMP-7 increases the expression pattern for angiogenic and inflammable cytokines: A matched pair analysis. J. Inflamm. Res..

[19] Masquelet A.C., Begue T. (2010). The concept of induced membrane for reconstruction of long bone defects. Orthop. Clin. N. Am..

[20] Moghaddam-Alvandi A., Zimmermann G., Büchler A., Elleser C., Biglari B., Grützner P.A., Wölfl C.G. (2012). Results of nonunion treatment with bone morphogenetic protein 7 (BMP-7). Unfallchirurg.

[21] Pelissier P., Masquelet A.C., Bareille R., Pelissier S.M., Amedee J. (2004). Induced membranes secrete growth factors including vascular and osteoinductive factors and could stimulate bone regeneration. J. Orthop. Res..

[22] Helbig L., Bechberger M., Aldeeri R., Ivanova A., Haubruck P., Miska M., Schmidmaier G., Omlor G.W. (2018). Initial peri- and postoperative antibiotic treatment of infected nonunions: Results from 212 consecutive patients after mean follow-up of 34 months. Ther. Clin. Risk Manag..

[23] Dapunt U., Spranger O., Gantz S., Burckhardt I., Zimmermann S., Schmidmaier G., Moghaddam A. (2015). Are atrophic long-bone nonunions associated with low-grade infections?. Ther. Clin. Risk Manag..

[24] Gruber R., Koch H., Doll B.A., Tegtmeier F., Einhorn T.A., Hollinger J.O. (2006). Fracture healing in the elderly patient. Exp. Gerontol..

[25] Zura R., Braid-Forbes M.J., Jeray K., Mehta S., Einhorn T.A., Watson J.T., della Rocca G.J., Forbes K., Steen R.G. (2017). Bone fracture nonunion rate decreases with increasing age: A prospective inception cohort study. Bone.

[26] Hernandez R.K., Do T.P., Critchlow C.W., Dent R.E., Jick S.S. (2012). Patient-related risk factors for fracture-healing complications in the United Kingdom General Practice Research Database. Acta Orthop..

[27] Taormina D.P., Shulman B.S., Karia R., Spitzer A.B., Konda S.R., Egol K.A. (2014). Older age does not affect healing time and functional outcomes after fracture nonunion surgery. Geriatr. Orthop. Surg. Rehabil..

[28] Gortler H., Rusyn J., Godbout C., Chahal J., Schemitsch E.H., Nauth A. (2018). Diabetes and Healing Outcomes in Lower Extremity Fractures: A Systematic Review. Injury.

[29] Shibuya N., Humphers J.M., Fluhman B.L., Jupiter D.C. (2013). Factors associated with nonunion, delayed union, and malunion in foot and ankle surgery in diabetic patients. J. Foot Ankle Surg..

[30] Brinker M.R., O’Connor D.P. (2007). Outcomes of tibial nonunion in older adults following treatment using the Ilizarov method. J. Orthop. Trauma.

[31] Murena L., Canton G., Vulcano E., Surace M.F., Cherubino P. (2014). Treatment of humeral shaft aseptic nonunions in elderly patients with opposite structural allograft, BMP-7, and mesenchymal stem cells. Orthopedics.

[32] Bishop J.A., Palanca A.A., Bellino M.J., Lowenberg D.W. (2012). Assessment of compromised fracture healing. J. Am. Acad. Orthop. Surg..

